# Bioactive Secondary Metabolites of Marine Fungi 2.0: Expanding Chemical Diversity Through Ecological and Cultivation-Based Discovery

**DOI:** 10.3390/md24100346

**Published:** 2026-09-30

**Authors:** Hee Jae Shin

**Affiliations:** 1 Marine Natural Products Chemistry Laboratory, Korea Institute of Ocean Science and Technology, 385 Haeyang-ro, Yeongdo-gu, Busan 49111, Republic of Korea; shinhj@kiost.ac.kr; 2 Department of Marine Technology and Convergence Engineering, University of Science and Technology, 217 Gajungro, Yuseong-gu, Daejeon 34113, Republic of Korea

Marine-derived fungi occupy ecologically distinctive niches and have long been recognized as prolific sources of structurally unique and biologically active secondary metabolites. Their chemical output reflects not only a substantial biosynthetic capacity but also adaptation to environmental and biological interactions in sediments, mangroves, sponges, cold seeps, and other marine-associated habitats. Consequently, marine fungal natural product research continues to contribute new molecular scaffolds while also providing opportunities to investigate how ecological context and cultivation conditions influence secondary metabolism [1,2,3].

This Special Issue, “Bioactive Secondary Metabolites of Marine Fungi 2.0”, has brought together seven papers that illustrate these complementary dimensions of the field. The collection encompasses the isolation and structural elucidation of new metabolites; the evaluation of antibacterial, antifungal, insecticidal, and antinociceptive activities; a focused review of marine-derived antifungal agents; and the use of fungal co-cultivation to induce additional secondary chemistry. The papers are referred to below as Contributions 1–7 and are listed separately in the List of Contributions; general literature citations are given in square brackets and correspond to the References section. The Special Issue website can be found here: https://www.mdpi.com/journal/marinedrugs/special_issues/U82S20H572 (accessed on 25 August 2026).

An initial theme that emerges from the collection is that ecologically targeted strain selection remains an effective entry point for chemical discovery. In Contribution 1, Lee et al. characterized meirols A–C from the marine-derived fungus *Meira* sp. 1210CH-42, thereby expanding the structural space of fungal catecholic metabolites. In Contribution 2, Arias Cardona et al. investigated the sponge-associated *Aspergillus fischeri* MMERU23 and identified hydroxytakakiamide together with other constituents; selected metabolites were subsequently evaluated in an antinociceptive model, providing a direct link between chemical characterization and pharmacological assessment. In Contribution 3, Che et al. examined *Aspergillus subversicolor* CYH-17 from the Haima cold seep and elucidated new phenol derivatives using spectroscopic methods, ECD calculations, and X-ray crystallography. Taken together, these studies reinforce the continuing value of carefully selected marine habitats and host associations as sources of chemically distinct fungal strains.

A second theme is the breadth of biological functions represented by marine fungal metabolites. In Contribution 4, Hang et al. reviewed marine-derived metabolites with antifungal activity and organized the field according to structural class, cellular target, and mechanism of action. Such a synthesis is particularly relevant because antifungal discovery requires not only potency but also selectivity and mechanistic differentiation from existing agents. In Contribution 5, Wang et al. reported six new metabolites from *Talaromyces minnesotensis* BTBU20220184 and showed that several compounds enhanced antibacterial activity against *Staphylococcus aureus* in combination with methicillin. In Contribution 6, Wang et al. described avellanins D–G from the mangrove-derived *Aspergillus fumigatus* GXIMD 03099, with selected metabolites displaying insecticidal and antibacterial activities. These contributions illustrate how structural novelty gains greater significance when it is accompanied by biologically informative evaluations.

The most conceptually distinct contribution concerns the deliberate perturbation of fungal metabolism. In Contribution 7, Wu et al. co-cultivated the mangrove endophytic fungi *Phomopsis asparagi* DHS-48 and *Phomopsis* sp. DHS-11 and identified three new metabolites among the induced products. The work demonstrated that interspecies interactions can alter the observable secondary-metabolite profile and reveal chemistry that may not be readily detected under routine monoculture conditions. Importantly, such experiments should be interpreted as the induction of secondary metabolic output unless direct transcriptional or genetic evidence establishes the activation of a particular biosynthetic gene cluster. Co-cultivation therefore complements the One Strain–Many Compounds (OSMAC) strategy and can be strengthened by LC–HRMS/MS-based metabolomics and molecular networking, which facilitate the dereplication and prioritization of induced molecular features [4,5,6].

Viewed as a whole, this Special Issue captures an important stage in the evolution of marine fungal natural product discovery. Classical isolation and rigorous structure elucidation remain indispensable, but the efficiency of these approaches can be improved by the ecological selection of strains, systematic modification of cultivation conditions, and early analytical prioritization. The seven contributions also emphasize that “chemical diversity” should not be treated as an end point. Rather, newly discovered compounds should be advanced, where justified, toward orthogonal biological assays, selectivity analysis, mechanism-of-action studies, and structure–activity relationships.

Several challenges remain. The re-isolation of known metabolites continues to consume time and biological material, underscoring the importance of dereplication before large-scale fermentation and purification. Low titers and culture-dependent metabolite production can also restrict biological characterization. For promising metabolites, optimization of culture conditions, re-fermentation, pathway-level studies, and, where feasible, semisynthetic or synthetic access should therefore be considered at an earlier stage. Equally important is the preservation of strain provenance, analytical metadata, and reproducible cultivation information so that biological observations can be traced to chemically and biologically defined material.

In summary, the papers collected in “Bioactive Secondary Metabolites of Marine Fungi 2.0” demonstrate that marine-derived fungi remain productive sources of structurally diverse and biologically active metabolites. More specifically, this collection shows how habitat-guided exploration and cultivation-based modulation can operate alongside conventional natural product chemistry to broaden the observable chemical space of marine fungi. Continued integration of these approaches with high-resolution analytical methods, rigorous dereplication, and deeper pharmacological validation should further enhance the value of marine fungal metabolites as chemical probes and as starting points for therapeutic and biotechnological development.

As Guest Editor, I sincerely thank all authors for their valuable contributions, the reviewers for their careful and constructive evaluations, and the editorial staff of *Marine Drugs* for their continuous support throughout this Special Issue. I hope that this collection will encourage further integration of marine microbial ecology, natural product chemistry, and pharmacology, and will stimulate the discovery of biologically meaningful metabolites from marine-derived fungi.
